# Preliminary Outcomes of an Ecological Momentary Intervention for Social Functioning in Schizophrenia: Pre-Post Study of the Motivation and Skills Support App

**DOI:** 10.2196/27475

**Published:** 2021-06-15

**Authors:** Daniel Fulford, David E Gard, Kim T Mueser, Jasmine Mote, Kathryn Gill, Lawrence Leung, Jessica Mow

**Affiliations:** 1 Department of Occupational Therapy Boston University Boston, MA United States; 2 Department of Psychological & Brain Sciences Boston University Boston, MA United States; 3 Department of Psychology San Francisco State University San Francisco, CA United States; 4 Department of Occupational Therapy Tufts University Somerville, MA United States

**Keywords:** schizophrenia, psychosis, social functioning, social skills, motivation, mHealth, smartphone, mobile phone

## Abstract

**Background:**

People with schizophrenia and other serious mental illnesses often lack access to evidence-based interventions, particularly interventions that target meaningful recovery outcomes such as social functioning and quality of life. Mobile technologies, including smartphone apps, have the potential to provide scalable support that places elements of evidence-based interventions at the palm of patients’ hands.

**Objective:**

We aim to develop a smartphone app—called Motivation and Skills Support—to provide targeted social goal support (eg, making new friends and improving existing relationships) for people with schizophrenia enrolled in a stand-alone open trial.

**Methods:**

In this paper, we presented preliminary outcomes of 31 participants who used the Motivation and Skills Support app for 8 weeks, including social functioning pre- to postintervention, and momentary reports of treatment targets (eg, social motivation and appraisals) during the intervention.

**Results:**

The findings suggest that the intervention improved self-reported social functioning from baseline to treatment termination, particularly in female participants. Gains were not maintained at the 3-month follow-up. Furthermore, increased social functioning was predicted by momentary reports of social appraisals, including perceived social competence and the extent to which social interactions were worth the effort.

**Conclusions:**

The implications of these findings and future directions for addressing social functioning in schizophrenia using mobile technology have been discussed.

**Trial Registration:**

ClinicalTrials.gov NCT03404219; https://clinicaltrials.gov/ct2/show/NCT03404219

## Introduction

### Background

For people with schizophrenia and other serious mental illnesses, psychosocial interventions demonstrate efficacy in improving important recovery outcomes, including occupational and social functioning [1]. In particular, negative symptoms such as low drive can improve through individual and group-based behavioral approaches such as social skills training (SST) and cognitive behavioral therapy for psychosis (CBTp) [2-4]. Although antipsychotic medications are the first line of treatment for both acute psychotic symptoms and prevention of relapses in schizophrenia [5], evidence-based psychosocial approaches can effectively address impairments in functioning that persist despite adequate pharmacological treatment [6,7].

Despite the demonstrated efficacy of psychosocial approaches to care, most individuals with schizophrenia experience long-standing challenges with social impairment [8]. Limited access to evidence-based care and the need for regular support beyond the time-limited nature of existing treatment for people with resource limitations contribute to poor long-term outcomes for these patients [9,10]. Effects of CBTp on functioning, for example, often do not persist far beyond treatment termination [11]. Recent mobile approaches to psychosocial support, such as smartphone apps, can help increase access to evidence-based, ongoing support. This is particularly important now, given increasing smartphone ownership among those with serious mental illnesses [12,13].

App-based interventions also have other advantages beyond in-person treatment. Apps can support real-time self-management outside of the therapy office, which may be particularly useful for addressing the dynamic nature of concerns that can arise during social interactions. Recently developed app-based interventions have shown promise in addressing psychosocial outcomes in a mobile format. For example, the FOCUS smartphone app is a multifaceted mobile intervention that targets auditory hallucinations, mood, sleep, functioning, and medication adherence [14]. FOCUS has demonstrated similar efficacy to standard in-person psychosocial approaches to care for people with schizophrenia, including in reducing symptoms and improving broadly defined recovery outcomes [15]. Other smartphone apps have been designed to address motivational impairment more directly, including in patients with early psychosis [16]. Text message–based approaches incorporating goal setting and other cognitive behavioral elements have also been used to target motivational negative symptoms [17,18]. Granholm et al [19] also recently developed a mobile app called CBT2go to supplement in-person CBTp.

Although existing mobile interventions address the broad needs of individuals with psychotic disorders in a comprehensive approach—including in combination with ongoing face-to-face care—they were not designed to directly target the multifaceted social needs of people with psychotic disorders, often described as the most critical need among patients themselves [20]. Indeed, only one of the reviewed studies showed improvements in social functioning, an effect that did not persist beyond treatment termination [19]. Thus, there remains a need for mobile approaches to directly target the complex, multifaceted nature of impaired social functioning in psychotic disorders.

### Objectives

To address this need, we aim to develop the Motivation and Skills Support (MASS) smartphone app. Our primary aims are to (1) develop an ecological momentary intervention (EMI) that integrates evidence-based psychosocial treatment approaches with insights from basic affective, motivational, and cognitive science to target key contributors to impaired social functioning in schizophrenia (namely, social motivation); (2) translate elements of SST and CBTp to allow for real-time support of social goal attainment in people’s daily lives; (3) systematically enhance social motivation by providing feedback of information about prior affective social experiences related to individuals’ social goals; and (4) incorporate stakeholder input (people with schizophrenia and expert clinicians) in the design of the intervention to assess attitudes regarding mobile technology and include content relevant to the everyday social needs of people with schizophrenia (see Fulford et al [21] for a more detailed description of app development, including data on usability and acceptability).

In this paper, we describe the preliminary outcomes of the MASS app in an open pilot trial. We report changes in clinical outcomes from baseline to treatment termination and 3-month follow-up. We also report changes in key intervention targets (ie, social motivation, associated behavior, and appraisals) during the intervention, as reported by ecological momentary assessment (EMA). We hypothesized that social functioning would increase and positive and negative symptoms would decrease during the intervention and that changes would be maintained at follow-up. We also predicted significant improvements in EMA-reported treatment targets throughout the intervention and that these targets would be associated with improvements in clinical outcomes from baseline to termination.

## Methods

### Study Procedures

This was a two-site, open pilot intervention study that took place in the Boston and San Francisco Bay areas (registered clinical trial NCT03404219). Clinical and outcome data were collected on three occasions: baseline (preintervention), at the end of the 8-week intervention (treatment termination), and 3 months following treatment termination (follow-up). Participants were first screened over the phone; those who met the screening criteria were invited to complete a baseline session that started with informed consent, followed by an assessment of eligibility and clinical measures. This was followed by an intervention period. At the end of each assessment (baseline, termination, and follow-up), participants were reimbursed for their time with cash payments.

### Participants

We recruited people with schizophrenia using fliers and word-of-mouth at community treatment and rehabilitation centers that serve people with serious mental illness. The inclusion criteria were a diagnosis of schizophrenia between the ages of 18 and 65 years, receiving current pharmacological treatment, psychotherapy, or both and fluency in English. Exclusion criteria were current (past 6 months) substance use disorder, self-reported current suicidal ideation (via structured diagnostic interview), or self-reported diagnosis of a neurological disorder. Although the study was advertised as potentially of interest to those who wished to improve their social lives, social impairment or interest in improving social functioning were not formal inclusion criteria.

### Intervention: MASS Mobile App

At the baseline assessment, participants worked with a research assistant to collaboratively select a social goal they wanted to work on, which should be feasible to achieve during the intervention. This social goal was programmed into the app by the research assistant, and then participants were introduced to the smartphone and the app, and they were asked to demonstrate basic phone and app functions to ensure comprehension and identify any potential challenges. Participants were provided with these smartphones to keep (Samsung Galaxy S8), and prepaid data and call and text plans were provided during the intervention period. Participants could access the app at any time during the intervention and could also access the SST video content beyond the study through links on the phone’s home screen.

Intervention content was delivered twice daily, 7 days a week for 60 days, via automatic push notifications in concert with the EMA surveys—one in the morning and one in the evening, using semirandom administrations (ie, within blocks of 2.5 hours). We did not reimburse participants to respond to surveys and encouraged the use of the app on an as-needed basis for social goal support. During the intervention period, research assistants attempted to contact participants once weekly (or less frequently if the participant preferred) through brief check-in phone calls to address any technical issues or other questions regarding the intervention. When these check-ins occurred, they were generally no longer than 10 minutes and focused exclusively on technical support for the phone or app, given that some participants did not have a history of smartphone ownership or experience with apps.

**Figure 1:**
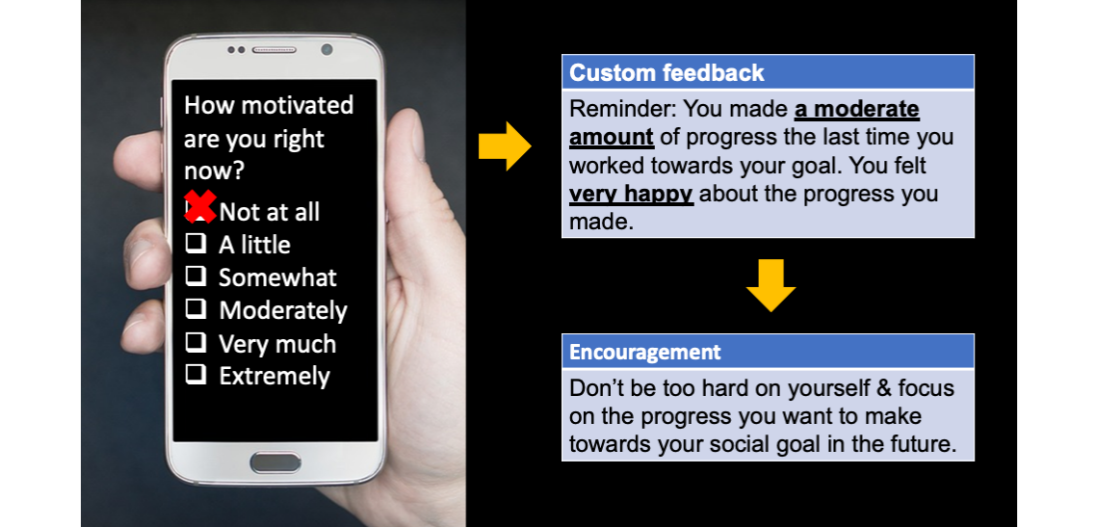
Motivation and Skills Support app content: feedback and validation and encouragement to address social motivation impairment.

Broadly, the intervention was developed as a targeted approach for addressing social functioning (primary outcome) in people with schizophrenia in a scalable mobile format. We used the Ethica Data mobile platform to deliver the intervention content through a native Android operating system app. The key elements included SST to address social skill challenges as they relate to goal progress in daily life and structured social goal support and feedback to address social motivation impairments that interfere with forming and maintaining social bonds, which were the primary targets of the intervention. Elements of cognitive behavioral therapy—including self-monitoring of progress toward social goals and social appraisals (ie, perceptions of social competence in recent interactions), and then feedback based on participants’ own ratings from previous EMA instances—were incorporated to address these social motivation impairments. For example, when participants reported low motivation to work toward their social goal, the app would remind them of positive social experiences that occurred in earlier interactions (Figure 1). These components were informed by models of motivational impairment that suggest challenges in accessing memories of prior positive experiences can interfere with the drive to attain goals [22-24]. The app also provides validating and encouraging statements to increase anticipatory pleasure for future social experiences, consistent with the temporal experience of the pleasure model of impairment in schizophrenia [25]. Details on the iterative development process and theoretical framework for the MASS app are provided in a recent paper by our team [21].

The format of the MASS app was an EMI, which consisted of push notifications via a smartphone that linked the user to app content related to social goal support and feedback. SST content included social goal support, including a list of specific steps the user could choose from and video content designed to provide modeling of effective communication in real-world contexts. The videos included two pairs of actors (1 man and 1 woman in each) demonstrating eight primary social skills (eg, active listening, sharing pleasant or unpleasant feelings), mirroring those covered in SST (links to SST videos are provided in Multimedia Appendix 1) [26]. All skills were demonstrated in a full-length video that included narratives. Participants were instructed to watch the full-length video on their own time during the first week of the intervention; full-length videos were accessible on the smartphone’s home screen. The video narratives included two stories: one involved 2 old friends reconnecting after several years apart, and the other included 2 acquaintances working toward developing a stronger connection. Although not explicitly stated for all characters, the subtext was that each narrative included a character with a history of mental health challenges; as such, disclosing mental health needs was one demonstrated skill. During the intervention, SST videos were delivered in short clips embedded within the EMI content, structured based on which skills were displayed.

The MASS app was personalized during an orientation meeting with the participant so that the goal support was specific to each participant’s identified social goal selected from a list of available goals curated by the research team. For example, if the participant chose to *make a new friend by going to events* as their desired goal at the baseline assessment**,** during the intervention period, the app would provide a list of steps required to attain that specific goal from which the participant could select (eg, “Identify an event that interests you by searching online or in a newspaper/publication,” “Find transportation to an event [eg, ask a relative to drive you, or look up the train, subway, or bus schedule],” or “Introduce yourself to someone at an event and start a conversation by saying hello”). This content is provided in the context of EMI delivered via notifications twice daily throughout the intervention period. Each step included the option to view additional information about how to complete the step and provided specific examples when appropriate (eg, if a participant selected “Identify an event that interests you,” the app offered specific websites as suggestions). After being reminded of their primary social goal, the participant had the option of selecting one of the available steps to work on during the next several hours. If the participant elected to work on one of the suggested steps at that time, the EMI would query the participant as to how motivated they were to work toward that step and how much progress they anticipated making on the step (Figure 1). In the final section of the survey, the participant had the opportunity to view SST content videos, with guidance as to which videos would be most relevant, given which step the participant chose to work on related to their goal. In the abovementioned example, the participant could select SST video clips that demonstrated effective ways of using active listening and/or finding common interests.

For instances in which participants either elected to not work on their social goal or when they elected to work on their goal but reported low motivation and/or anticipated progress, they were provided a statement of validation (eg, “Everyone has difficulties working towards their goals sometimes”) and encouragement (eg, “We find that the best way to think of social goals and social skills is that you are building a bridge, brick by brick, day by day. This takes time and effort”) randomly administered by the app from a set of 31 potential statements (16 validation and 15 encouragement). These statements were generated by our research team, which includes 4 clinical psychologists with extensive experience working with people with schizophrenia. The participants also had the opportunity to view SST content videos at any time, regardless of whether they wanted to work on their social goals at that time.

### Measures

#### Primary Outcome: Social Functioning

The primary outcome measures for this study were the Social Functioning Scale (SFS [27]) and the Heinrichs Quality of Life Scale–Interpersonal Relations (QLS-IR [28]) subscale. The SFS is a self-report instrument designed to capture a wide range of socially relevant behaviors and activities and is the most widely used scale for assessing social functioning in schizophrenia [29]. The 79-item scale includes assessment of social competence and performance across seven domains: withdrawal (social isolation), interpersonal functioning (social contacts and interpersonal competence), prosocial activities (social recreation activities), recreation (solitary recreation activities), independence‐competence (social abilities), independence‐performance (actual performance of social skills), and employment. Standard scores were calculated, with a mean of 100 (SD 15) across all domains, and the total score was computed based on the mean of all domains. Lower scores represent more impairment in social functioning. The QLS-IR is an interview-rated measure that includes items designed to address social functioning across a range of social contacts and relationships throughout the past 30 days.

The QLS-IR (along with other domains assessed by the QLS, including role functioning and motivation) was originally designed to measure impairments reflective of the deficit syndrome (ie, persistent, primary negative symptoms), but the scale has been used extensively as an outcome measure in intervention studies [30]. Although the SFS captures more subjective qualities of interpersonal functioning (ie, satisfaction and perceived competence), the QLS-IR assesses both the quantity and quality of social functioning as ascertained by a trained interviewer. As such, we included both assessments as primary outcome measures to gather data on the extent to which the MASS app intervention addressed the broad spectrum of social functioning.

#### Secondary Outcomes: Positive and Negative Symptoms

We used the Brief Psychiatric Rating Scale [31] as our measure of positive symptoms. We administered the following items: somatic concern, grandiosity, suspiciousness, hallucinations, unusual thought content, bizarre behavior, disorientation, and conceptual disorganization [32,33]. For negative symptoms, we used the Clinical Assessment Interview for Negative Symptoms (CAINS) [34]. The CAINS assesses experiential (motivation and pleasure [MAP]) and expressive deficits. We only present findings for the MAP items here, given that we did not expect the intervention to improve expressive deficits. Trained interviewers conducted these symptom assessments.

#### Treatment Target: Social Motivation (Desire, Behavior, and Interaction Appraisals)

EMA items addressed multiple components of social motivation, including the desire for social contact, the number of recent social interactions, and appraisals of the outcomes of these interactions. The desire for social contact was assessed with the following question: “How much would you like to talk to or interact with someone right now?” with response options ranging from 1 (not at all) to 5 (extremely). When participants elected to work on their social goal, they were asked, “How motivated are you to work on this step?” with response options ranging from 1 (not at all motivated) to 5 (as motivated as possible). The number of interactions was assessed with the following question: “How many conversations did you have online, by phone/text, or in person, since the last time you filled out a survey?” Response options included *none*, *1*, *2*, or *3 or more*.

We asked participants to rate their appraisals of the outcomes of recent interactions after instances in which they reported having a recent conversation. These included items that addressed perceived social skills (“How well do you think you communicated in those conversations?” with response options ranging from 1 [I did not communicate well at all] to 4 [I communicated very well]), the extent to which interactions were worth the effort (“To what extent were those interactions worth the effort?” with response options ranging from 1 [not worth the effort at all] to 4 [definitely worth the effort]), and how likable they perceived themselves to be (“What do you think other people thought of you in those conversations?” with response options ranging from 1 [very unlikable] to 4 [very likable]).

### Analyses

We first examined distributions of the outcome measures to assess normality. We also ran bivariate correlations between the sample characteristics and clinical assessments to examine potential confounding factors. Assuming normal distributions, we ran separate repeated measures general linear models with each outcome variable as the dependent variable across the three time points (baseline, termination, and follow-up), including relevant covariates. We present tests of linear change and include effect size estimates (partial eta squared) for each outcome. We followed up general linear models with two-tailed paired-samples *t* tests to identify changes from baseline to treatment termination and present Cohen *d* effect sizes to estimate the magnitude of the short-term treatment effect.

For EMA data, we first examined the distributions of the variables of interest. For normally distributed variables, we ran mixed-effects linear models to examine developmental changes in each variable during the 8-week intervention period in separate models. We included the time point as a predictor of each EMA variable.

We also examined the extent to which the intervention target (ie, EMA-reported social motivation) predicted changes in social functioning from baseline to treatment termination. We included any EMA-reported variables that demonstrated a linear change during treatment and used the covariate method to demonstrate changes in social functioning via regression models, with the aggregated EMA variables as predictors, baseline clinical measures as covariates, and termination measures as outcomes [35]. We also included gender as a covariate in analyses of social functioning, given well-documented gender differences in social skills and other social outcomes in schizophrenia [36].

## Results

### Overview

**Figure 2:**
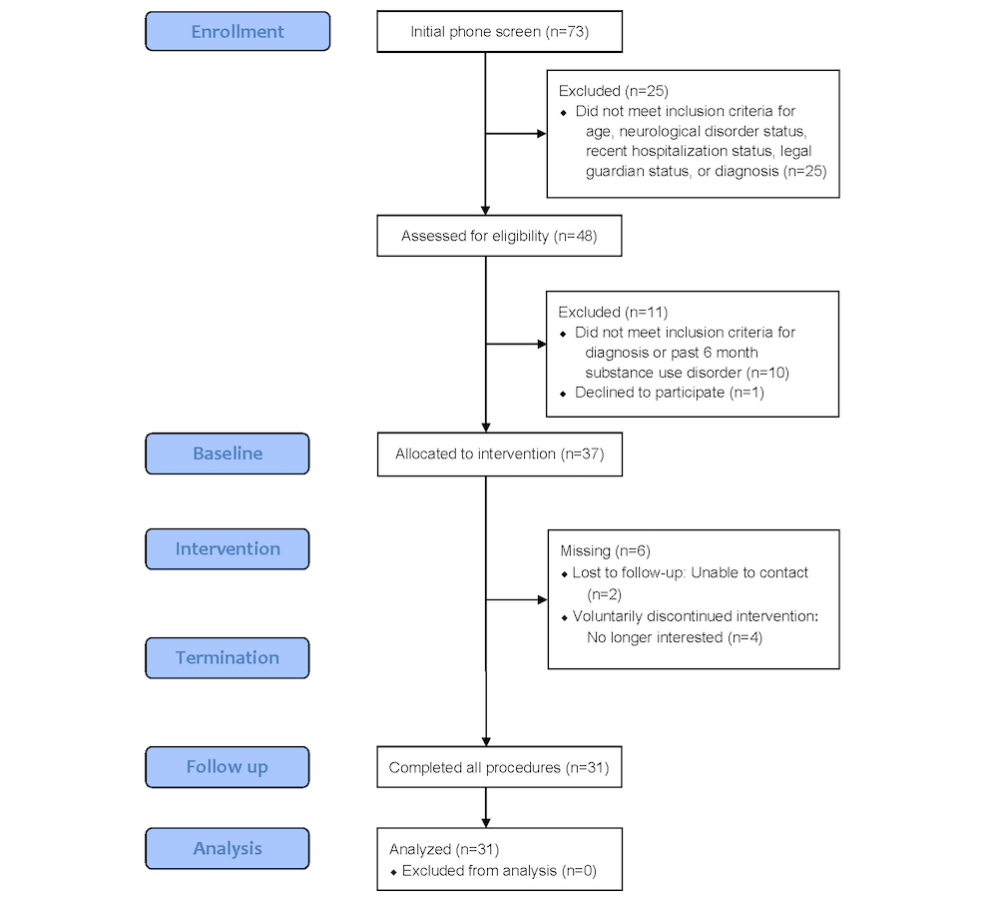
CONSORT (Consolidated Standards of Reporting Trials) chart for the Motivation and Skills Support app open pilot.

In total, 7 participants completed the study prematurely. Reasons for discontinued participation included no longer being interested in the study after initially enrolling, encountering technical difficulties with the mobile app, or being lost to follow up (Figure 2). This left 31 participants who completed the entire study. The sample characteristics are listed in Table 1. The final sample ranged in age from 22 to 65 years (median 48 years). The sample consisted of roughly half men, half schizophrenia, and half schizoaffective disorder and was racially diverse (17/31, 55% non-White). Most of the sample was unemployed, currently receiving disability payments, noncollege graduates, and never married.

Participants selected a broad range of social goals, including friends, family, and romantic relationships (see Table 2 for the selected goals). After treatment termination, we conducted an exit interview and, of the 28 participants, 19 (68%) reported completing some (10/28, 36%) or all (9/28, 32%) of their social goals (we did not collect these data for our first 3 participants). As one goal in particular—*practice conversation skills with an existing friend*—was potentially easier to achieve than the remaining goals, we tested whether goal attainment rates were higher for this goal than the others. Of the 5 participants who selected this goal, 4 (80%) met at least a part of their goal. Of the 23 participants who selected one of the remaining goals, 15 (65%) reported meeting at least part of their goals. These proportions were not significantly different (*X*^2^_1_=0.3; *P*=.60).

Participants responded to 42.5% (51/120) of push notifications on average, which was acceptable given the nature of the study (ie, the app was designed to be used on an as-needed basis for social goal support). There was a wide range of engagement in SST video content. One participant never watched the SST videos, whereas, on the other extreme, one participant viewed the videos 422 times. After removing this outlier, participants viewed the videos an average of 13.5 (SD 14.52) times during the 60-day intervention period (median 10).

**Table 1:** Participant demographics and sample characteristics (n=31).

Variable			Values
**Diagnosis, n (%)**			
	Schizophrenia	17 (55)	
	Schizoaffective disorder	14 (45)	
**Race, n (%)**			
	Asian American	9 (29)	
	Black or African American	5 (16)	
	White	14 (45)	
	Multiracial	3 (10)	
	Hispanic or Latinx ethnicity	1 (3)	
Age (years), mean (SD)			46 (11)
Illness duration (years), mean (SD)			23.03 (12.52)
Gender (men), n (%)			16 (52)
Antipsychotic medication, n (%)			26 (87)
Marital status (married, cohabitating, or divorced), n (%)			4 (13)
Education (college graduate), n (%)			9 (29)
Employed (full- or part-time), n (%)			11 (36)
Disability (receive disability payments), n (%)			21 (75)
Smartphone history, n (%)			26 (87)

**Table 2:** Social goals selected and number completed (consider putting goals in order of most to least common; n=31).

Social goal	Participant, n (%)
Make a new friend by going to events	7 (23)
Make a new friend at work	1 (3)
Practice conversation skills with an existing friend	5 (16)
Reconnect with an old friend	2 (7)
Improve an existing friendship with regular activity	1 (3)
Improve relationship with family member you don’t live with or see regularly	7 (23)
Improve relationship with family member you live with or see regularly	3 (10)
Start a romantic relationship with someone you already know	1 (3)
Start a romantic relationship through a dating website	4 (13)
Participants who completed part or all of social goal	19 (68)^a^

^a^n=28 participants with data on goal attainment.

### Clinical Outcomes

Before analyzing clinical treatment outcomes, we examined potential differences in changes in social functioning (SFS and QLS-IR) from baseline to treatment termination based on sample characteristics, including gender (all but 1 participant identified as a man or a woman), age, race or ethnicity, and employment status (because the vast majority were never married, we did not examine this variable). There was only one difference among these variables: women demonstrated significantly greater improvement in SFS scores from baseline to treatment termination than men (*t*_30_=−2.19; *P*=.04; Cohen *d*=0.8).

#### Primary Outcome: Social Functioning

**Figure 3:**
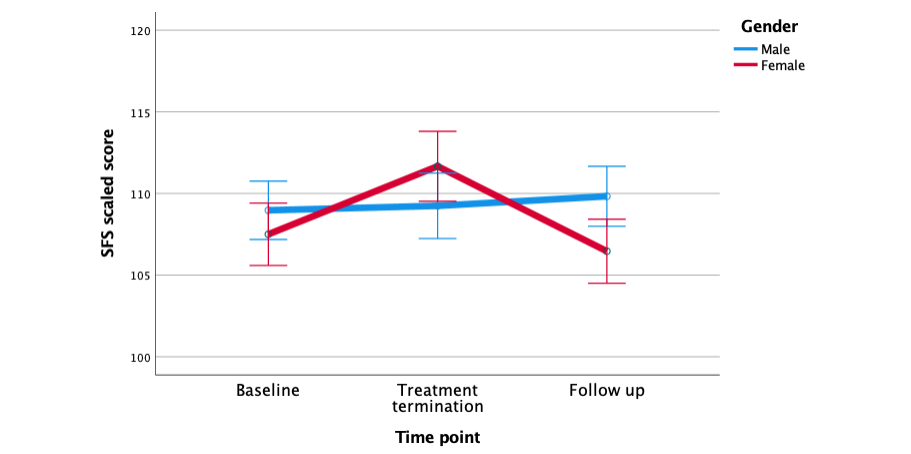
SFS by gender. SFS scores significantly differed from baseline to treatment termination among female participants (*P*=.04). SFS: social functioning outcomes.

The intervention outcomes are shown in Table 3. The mean SFS scores showed a moderate increase from baseline (107.99, SD=7.13) to treatment termination (110.13, SD 7.95; *t*_30_=−2.52; *P=*.02; Cohen *d=*0.44), but then decreased at follow-up (108.23, SD 7.29; overall *F*_2_*=*2.56; *P=*.09; partial η^2^=0.08). In an analysis of covariance model with gender as a between-subjects factor, the time by gender interaction effect was significant (*F*_2_*=*3.26; *P=*.046; partial η^2^=0.1). Women showed increases in SFS scores from baseline to termination, which then decreased from termination to follow-up. On the other hand, men showed relatively little change in SFS scores across the 3-time points (Figure 3). This time by gender interaction was best represented by a quadratic term (*F*_1_*=*7.55; *P*=.01; partial η^2^=0.21). The mean QLS-IR scores showed a small increase from baseline (2.60) to treatment termination (2.90; *t*_30_=−1.33; *P*=.19; Cohen *d=*0.24), and then a decrease at follow-up (2.70; overall *F*_2_*=*1.13; *P=*.33; partial η^2^=0.04).

**Table 3:** Intervention outcomes: social functioning, positive symptoms, and motivation and pleasure negative symptoms.

Measure	Baseline, mean (SD)	Treatment termination, mean (SD)	3-month follow-up, mean (SD)	Baseline to follow-up		Baseline to termination		Cohen *d*
				*F* test (*df*)	*P* value	Post hoc *t* test (*df*)	*P* value	
SFS^a^ total	108.00 (7.13)	110.13 (7.95)	108.23 (7.29)	2.56 (2)	.09	−2.52 (30)	.02	0.44
QLS-IR^b^	2.60 (1.36)	2.90 (1.15)	2.70 (1.25)	1.13 (2)	.33	−1.33 (30)	.19	0.24
BPRS^c^ positive symptoms	2.44 (0.70)	2.22 (0.78)	2.14 (0.76)	3.74 (2)	.03	2.49 (30)	.02	0.45
CAINS-MAP^d^	1.64 (0.70)	1.71 (0.60)	1.71 (0.77)	0.19 (2)	.83	−0.65 (30)	.52	0.12

^a^SFS: Social Functioning Scale.

^b^QLS-IR: Quality of Life Scale–Interpersonal Relations.

**^c^**BPRS: Brief Psychiatric Rating Scale.

^d^CAINS-MAP: Clinical Assessment Interview for Negative Symptoms-Motivation and Pleasure.

#### Positive and Negative Symptoms

Brief Psychiatric Rating Scale positive symptoms decreased significantly across the 3 time points (overall, *F=*3.74; *P*=.03; partial η^2^=0.11). Post hoc tests revealed a moderate decrease from baseline to follow-up (*t*_30_=2.49; *P=*.02; Cohen *d=*0.45), and a small decrease from baseline to termination (*t*_30_=1.65; *P=*.11; Cohen *d=*0.30) and from termination to follow-up (*t*_30_=1.04; *P=*.31; Cohen *d=*0.19). CAINS-MAP scores did not change across the 3 time points (overall, *F*=0.19; *P=*.83; partial η^2^=0.01).

### Change in Momentary Treatment Targets and Associations With Social Functioning

As reported earlier, the missing data for the EMA surveys were 55%. Missing EMA data were unrelated to sample characteristics (eg, gender, age, or diagnosis) or clinical variables at baseline (eg, social functioning and negative symptoms) nor did it change over time (*b*=−0.002, SE 0.003; *X*^2^_1_=0.5; *P=*.50). Thus, the findings included all the participants in the study.

General desire to interact with others (*b=*−0.04, SE 0.05) and motivation to work on steps toward social goals (*b*=0.02, SE 0.08) did not change during the intervention (see Multimedia Appendix 1 for outputs). The number of interactions reported via EMA decreased across the 8-week intervention period (*b*=−0.11, SE 0.05; *t*_30_=−2.18; *P*=.03; 95% CI −0.20 to −0.01). The number of interactions reported showed a small, negative association with the number of missing surveys (*r*=−0.33; *P=*.09), suggesting that the decline in reported interactions could have reflected survey burden, or simply that those with fewer interactions were less likely to engage with the app.

**Figure 4:**
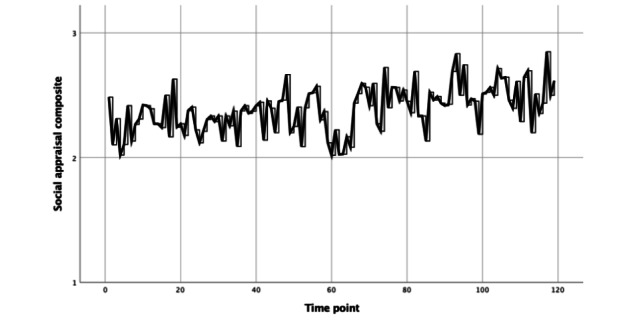
Changes in ecological momentary assessment-reported social appraisals during the intervention period.

A preliminary analysis revealed that the three items assessing social appraisals of recent interactions (how likable they were, how well they communicated, and how much these interactions were worth the effort) were all highly correlated with each other (*r*>0.80). As such, we calculated a composite social appraisal variable, which was the average of these three items (α=.95). This social appraisal composite increased significantly in the intervention period (*b*=0.11, SE 0.05; *t*_30_=2.23; *P*=.03; 95% CI 0.01 to 0.19; Figure 4).

We then examined the degree to which aggregated EMA-reported social appraisals predicted changes in social functioning (SFS and QLS-IR) from baseline to treatment termination using linear regression. We included gender as a covariate in these models. EMA measures of social appraisals predicted significant increases in SFS scores from baseline to treatment termination. Social appraisals were not associated with changes in QLS-IR scores (Table 4).

**Table 4:** Changes in social functioning (Social Functioning Scale and Quality of Life Scale–Interpersonal Relations) from baseline to treatment termination as predicted by ecological momentary assessment-reported social appraisals.

Social functioning		b (SE)	*t* test (*df*)	*P* value	*R^2^*	*R^2^* change
**SFS^a^** **termination**						
	SFS baseline	0.848 (0.124)	6.820 (30)	<.001	0.594	N/A^b^
	Gender	3.540 (1.701)	2.081 (30)	.047	0.650	0.056
	Social appraisal composite	2.810 (1.362)	2.063 (30)	.049	0.700	0.049
**QLS-IR^c^** **termination**						
	QLS-IR baseline	0.465 (0.135)	3.447 (30)	.002	0.321	N/A
	Gender	0.461 (0.356)	1.295 (30)	.21	0.362	0.041
	Social appraisal composite	0.031 (0.288)	0.108 (30)	.92	0.363	0.001

^a^SFS: Social Functioning Scale.

^b^N/A: not applicable.

^c^QLS-IR: Quality of Life Scale–Interpersonal Relations.

## Discussion

### Principal Findings

This paper presented the preliminary outcomes of an open pilot trial of the MASS smartphone app in 31 outpatients diagnosed with schizophrenia or schizoaffective disorder. Low study attrition and high levels of engagement in the app suggest that this stand-alone mobile intervention was feasible. In addition, the MASS mobile intervention demonstrated promise for addressing social needs in people with schizophrenia, with significant increases in our primary outcome, self-reported social functioning, as measured by the SFS, from baseline to treatment termination. This is the first study to demonstrate that a stand-alone mobile intervention can be effective at improving social functioning in schizophrenia, making these preliminary findings an important step forward in this important area of high need.

The significant improvement in self-reported social functioning may reflect increased satisfaction with and perceived competence in social interactions. This is evidenced by changes in key intervention targets reported by EMA in the 8-week intervention period. Participants reported significant increases in positive social appraisals, including perceptions of how well they thought they communicated with others, how likable they were, and the degree to which these interactions were worth the effort. Furthermore, the mean levels of these positive social appraisals were associated with significant increases in social functioning from baseline to treatment termination, suggesting that these appraisals show promise as key intervention targets.

Increases in positive social appraisals could have resulted from several components of the MASS app intervention. For example, SST video content and associated opportunities to practice skills could have increased participants’ confidence in their social abilities, leading to increased perceptions of effective communication skills. This content could also have led to more effective communication and the associated natural positive consequences of those skills. Perceptions of effective communication could also have resulted from support for social goals, such as regular reminders of specific steps required to attain desired social goals; feedback on goal progress (ie, completing such a step) could have provided concrete evidence of competence. This would be consistent with theories of goal pursuit, including those that form the foundation for organizing goals in ways that make them specific, measurable, attainable, realistic, and time-limited [37,38]. One way the MASS app could have been effective in improving perceived social effectiveness is by serving as a tool to structure larger social goals in ways that make them more attainable, thus increasing confidence in the ability to ultimately reach them.

A potential challenge for people with schizophrenia spectrum disorders is navigating the complexity of social interactions; such complexity may increase the perceived effort required for a successful interaction [39,40]. Thus, increased positive social appraisals could have also led to reductions in perceived effort required to communicate effectively or simply could have enhanced the extent to which these interactions were perceived as enjoyable. More work is needed to disentangle the specific mechanisms involved in modifying these appraisals.

It is also possible that features of the study other than MASS app content could have served to increase positive social appraisals and social functioning. Although the MASS app was delivered as a stand-alone mobile intervention, our research team did have contact with participants through check-ins over the phone (eg, troubleshooting technical issues and/or discussing any challenges with the intervention app). These check-ins could have had a positive effect on positive social appraisals, an unintended consequence reported in previous EMA work in schizophrenia [41]. It will be helpful to quantify the degree of social contact provided by research staff in future work examining the efficacy of digital interventions to better understand the extent to which this serves as an active intervention ingredient [9]. It is also possible that increased awareness of social activity associated with completing EMA surveys could have served to improve social functioning. For example, repeated monitoring and reporting of social contacts could have primed participants to reach out to others and increase communication, further increasing social functioning by the end of the intervention. However, the impact of awareness alone on overall social functioning was likely to be small relative to the active intervention components.

Improvements in self-reported social functioning were not maintained at the 3-month follow-up. The lack of sustained improvement indicates that ongoing support (or support for a longer period) may be needed to maintain positive social outcomes in people with schizophrenia, especially concerning attaining long-term goals (eg, making a new friend). This is not surprising given longitudinal work demonstrating significant stability in social impairment in this population throughout long periods [8,42] and work showing limited long-term gains in social functioning in the context of time-limited evidence-based interventions (eg, cognitive behavioral therapy) [11]. Important follow-up work should examine adequate dosing and/or the need for the long-term stability of support in social functioning outcomes.

Significant reductions in positive symptoms, particularly suspiciousness, hostility, and unusual thought content, suggest the promise of the MASS intervention in addressing auxiliary experiences associated with social functioning. Active intervention components, such as social goal support, could have served to reduce the potential for ambiguity in social interactions through increased structure and predictability. This structured support could have further reduced feelings of suspiciousness, given the higher likelihood of interpretation biases in ambiguous situations among people who experience paranoia [43]. To better understand this possibility, future work could incorporate momentary measures of interpretation biases (ie, perceiving others as threatening) to examine the extent to which these may have decreased in the intervention. In addition, given the lack of a control group in this study, it is possible that reductions in positive symptoms were because of the passage of time alone and not specific to the intervention. Although we did not recruit participants during a period of heightened symptoms (most were at the low end of the spectrum), it is still possible that those interested in participating were experiencing more distress than usual and that this dissipated naturally over time. However, it is important to note that nearly all participants were on a stable dose of antipsychotic medication at study entry.

There were differences in social functioning outcomes by gender: for women, SFS scores increased significantly from baseline to treatment termination but then returned to baseline levels at the 3-month follow-up assessment). For men, the SFS scores remained relatively unchanged across the three time points. Women with schizophrenia typically have better social functioning than men [44,45]. For example, women show better social skills [36,46] and are more likely to marry [47,48] than men with schizophrenia. However, differences observed in this study did not appear to be related to baseline levels of social functioning, as mean values were not significantly different between genders at baseline. Differences also did not appear to be related to adherence to the intervention (men and women did not differ in EMA surveys completed). Another possibility is that social functioning outcomes are related to the types of social goals selected. There was a noticeable difference in goals selected between genders: 5 men chose to work toward improving a relationship with a family member whom they did not live with or regularly see, whereas only 1 woman chose this goal. It is possible that this goal was more challenging than others; however, given the small sample size, we cannot definitely test this assumption. Nonetheless, in future work, it will be important to collect additional data on the extent to which goals vary in difficulty and how variance in difficulty might influence outcomes.

The lack of a positive impact of the MASS app on interview-rated social functioning (QLS-IR) or the number of social contacts (reported via EMA) suggests that although the intervention improved a broad range of perceived social outcomes (as assessed by the SFS), it did not impact specific markers of social functioning, such as the number of contacts or social network size. Scores on the QLS-IR reflect increases in social network size and degree of contact with relatives and acquaintances, among other objective outcomes, similar to EMA-reported interactions. These and other outcomes, including negative symptoms, may be particularly challenging to improve in a brief, remotely delivered intervention and may require additional support that cannot be delivered in such a platform. As such, a way to improve the effectiveness of the MASS app (as with other mobile interventions) would be as a supplemental, augmentative intervention to be used alongside standard evidence-based interventions delivered in standard, in-person contexts [9]. This approach was recently tested by Granholm et al [19], in which participants received up to 24 weeks of combined weekly group therapy and mobile intervention. Improvements in key targets, including defeatist attitudes and negative symptoms, have been reported. Similar to this study, the authors demonstrated improvements in social functioning, but only from baseline to treatment termination (not over follow-up). Another way to improve the effectiveness of the MASS app, as mentioned earlier, would be to increase the length of the *active* intervention period, providing support for a sufficient amount of time for participants to achieve their desired social goals fully.

### Conclusions

As reported earlier, the findings are limited to the uncontrolled nature of this pilot study. As such, we cannot rule out the possibility that outcomes could be owing simply to the passage of time. Furthermore, our sample was relatively small, and we may have been underpowered to detect the small effects of the intervention. Another potential limitation of this study is that we provided mobile devices and data plans to all participants, making it unclear whether this intervention approach would be scalable to individuals who lack the resources needed for sustained ownership of personal smartphones. Despite these limitations, the MASS app demonstrates promise as a stand-alone mobile intervention and is ultimately used as a supplemental intervention designed to address social functioning needs in people with schizophrenia.

## Appendix

Multimedia Appendix 1 Motivation and Skills Support app social skills training video content.

## Abbreviations

CAINS: Clinical Assessment Interview for Negative Symptoms
CBTp: cognitive behavioral therapy for psychosis
EMA: ecological momentary assessment
EMI: ecological momentary intervention
MAP: motivation and pleasure
MASS: Motivation and Skills Support
QLS-IR: Quality of Life Scale–Interpersonal Relations
SFS: Social Functioning Scale
SST: social skills training
