# Supplementary material for: Preliminary Outcomes of an Ecological Momentary Intervention for Social Functioning in Schizophrenia: Pre-Post Study of the Motivation and Skills Support App
Source: JMIR Ment Health. 2021 Jun 15;8(6):e27475. doi: 10.2196/27475 (PMC8277369; doi:10.2196/27475)
Supplement: Multimedia Appendix 1 [file mental_v8i6e27475_app1.pdf]

UPDATED MASS App pilot SST videos

Long form (combined G&M and J&S):
Part 1: https://www.youtube.com/watch?v=x5npKkQnKXM&feature=youtu.be
Part 2: https://www.youtube.com/watch?v=4pTC2NYolKA&feature=youtu.be
Part 3: https://www.youtube.com/watch?v=sAiuV3jYtcg&feature=youtu.be
Part 4: https://www.youtube.com/watch?v=bqJX-BONnfs&feature=youtu.be

Skill intros
1: https://www.youtube.com/watch?v=7rCtwxFNwwE
2: https://www.youtube.com/watch?v=tbIL1Om3ayg
3: https://www.youtube.com/watch?v=brhPoTcqjCk
4: https://www.youtube.com/watch?v=OYIuKKC9JYs
5: https://www.youtube.com/watch?v=ASOwtnQFAl8
6: https://www.youtube.com/watch?v=h5ITWIelJfg
7: https://www.youtube.com/watch?v=Izu_BTcFlC8
8: https://www.youtube.com/watch?v=-T0vJx3qmgo

Gabriel and Maria (re-mastered audio)
1C combined: https://www.youtube.com/watch?v=ZDV7lzhDsjg
1D Combined: https://www.youtube.com/watch?v=8uQvSW8sgqQ
2C combined: https://www.youtube.com/watch?v=vb6K2AxJgOA
3B combined: https://www.youtube.com/watch?v=_flNiAoYOyQ
3D combined: https://www.youtube.com/watch?v=Qn_kbtztUSo
4A combined: https://www.youtube.com/watch?v=dyjKf1LkLio
5A combined: https://youtu.be/see8oSd6FTI
5C combined: https://youtu.be/Um_CnHv8GJU
6A combined: https://youtu.be/_fkS7cqGQXs
7B combined: https://youtu.be/skYYIWwXCTE
7D combined: https://youtu.be/O5VV9koUHoo
8A combined: https://youtu.be/M0gVn2sRWV4
8C combined: https://youtu.be/hzC0Whw-HRw

Gabriel and Maria Midway Summary: https://youtu.be/F7wmIlygjBs
Gabriel and Maria Final Summary: https://youtu.be/H_IdZA0cZ5U

John and Sally

1A: https://www.youtube.com/watch?v=TmIbQ2jVBjw&feature=youtu.be
1B: https://www.youtube.com/watch?v=xC0dIbr_IPE&feature=youtu.be
2A: https://www.youtube.com/watch?v=arEPF--XM5Q&feature=youtu.be
2B: https://www.youtube.com/watch?v=chAoHF1SoQA&feature=youtu.be
3A: https://www.youtube.com/watch?v=MmFIzEItttc&feature=youtu.be
3C: https://www.youtube.com/watch?v=AIF33cDxmIU&feature=youtu.be
4B: https://www.youtube.com/watch?v=g-WTXESUtTw&feature=youtu.be
4C combined: https://www.youtube.com/watch?v=qdkW-1jMUbc&feature=youtu.be
5B: https://www.youtube.com/watch?v=VBrv68SX80g&feature=youtu.be
5D: https://www.youtube.com/watch?v=vw_D2nfP_B0&feature=youtu.be
6B: https://www.youtube.com/watch?v=YFLxZKHWqLQ&feature=youtu.be
6C: https://www.youtube.com/watch?v=KqPl-qy5tVQ&feature=youtu.be
7A: https://www.youtube.com/watch?v=Sd6j4QeQRno&feature=youtu.be
7C: https://www.youtube.com/watch?v=yAimds14XWg&feature=youtu.be
8B: https://www.youtube.com/watch?v=J4gaMV0T62Y&feature=youtu.be
8D: https://www.youtube.com/watch?v=8rSGkUEt3bA&feature=youtu.be

Active Listening:
Active Listening Intro: https://www.youtube.com/watch?v=7rCtwxFNwwE
Gabriel and Maria Part 1 https://www.youtube.com/watch?v=ZDV7IzhDsjg
Gabriel and Maria Part 2 https://www.youtube.com/watch?v=8uQvSW8sgqQ
John and Sally Part 1: https://www.youtube.com/watch?v=TmIbQ2jVBjw&feature=youtu.be
John and Sally Part 2https://www.youtube.com/watch?v=xC0dIbr_IPE&feature=youtu.be

Expressing Pleasant Feelings
Expressing Pleasant Feelings Intro: https://www.youtube.com/watch?v=tbIL1Om3ayg
Gabriel and Maria: https://www.youtube.com/watch?v=vb6K2AxJgOA
John and Sally Part 1: https://www.youtube.com/watch?v=arEPF--XM5Q&feature=youtu.be
John and Sally Part 2:
https://www.youtube.com/watch?v=chAoHF1SoQA&feature=youtu.be

Making Positive Requests
Making Positive Requests Intro: https://www.youtube.com/watch?v=brhPoTcqjCk
Gabriel and Maria Part 1: https://www.youtube.com/watch?v=_fINiAoYOyQ
Gabriel and Maria Part 2: https://www.youtube.com/watch?v=Qn_kbtztUSo
John and Sally Part 1: https://www.youtube.com/watch?v=MmFIzEItttc&feature=youtu.be

John and Sally Part 2: https://www.youtube.com/watch?v=AIF33cDxmIU&feature=youtu.be

Expressing Unpleasant feelings

Expressing Unpleasant Feelings Intro: https://www.youtube.com/watch?v=OYIuKKC9JYs
Gabriel and Maria: https://www.youtube.com/watch?v=dyjKf1LkLio
John and Sally Part 1: https://www.youtube.com/watch?v=g-WTXESUtTw&feature=youtu.be
John and Sally Part 2: https://www.youtube.com/watch?v=qdkW-1jMUbc&feature=youtu.be

Giving and Accepting Compliments

Giving and Accepting Compliments Intro: https://www.youtube.com/watch?v=ASOwtnQFAl8
Gabriel and Maria Part 1: https://youtu.be/see8oSd6FTI
Gabriel and Maria Part 2: https://youtu.be/Um_CnHv8GJU
John and Sally Part 1: https://www.youtube.com/watch?v=VBrv68SX80g&feature=youtu.be
John and Sally Part 2: https://www.youtube.com/watch?v=vw_D2nfP_B0&feature=youtu.be

Finding Common interests

Finding Common Interests Intro: https://www.youtube.com/watch?v=h5ITWIelJfg
Gabriel and Maria https://youtu.be/_fkS7cqGQXs
John and Sally Part 1:https://www.youtube.com/watch?v=YFLxZKHWqLQ&feature=youtu.be
John and Sally Part 2: https://www.youtube.com/watch?v=KqPl-qy5tVQ&feature=youtu.be

Compromising and Negotiating

Compromising and Negotiating Intro:https://www.youtube.com/watch?v=Izu_BTcFlC8
Gabriel and Maria Part 1: https://youtu.be/skYYIWwXCTE
Gabriel and Maria Part 2: https://youtu.be/O5VV9koUHoo
John and Sally Part 1: https://www.youtube.com/watch?v=Sd6j4QeQRno&feature=youtu.be
John and Sally Part 2: https://www.youtube.com/watch?v=yAimds14XWg&feature=youtu.be

Disclosing Mental Health Needs

Disclosing Mental Health Needs Intro https://www.youtube.com/watch?v=-T0vJx3qmgo
Gabriel and Maria Part 1: https://youtu.be/M0gVn2sRWV4
Gabriel and Maria Part 2: https://youtu.be/hzC0Whw-HRw
John and Sally Part 1: https://www.youtube.com/watch?v=J4gaMV0T62Y&feature=youtu.be
John and Sally Part 2: https://www.youtube.com/watch?v=8rSGkUEt3bA&feature=youtu.be
